# Linguistic and psychometric validation of a German version of the measure of moral distress for healthcare professionals (MMD-HP GER): results of a study with intensive care nurses

**DOI:** 10.1186/s12910-026-01522-3

**Published:** 2026-06-12

**Authors:** Larissa Forster, Sadhbh Byrne, Hannah Spielmann-Burkard, Susanne Weber, Madeleine von Strachwitz, Katja Kuehlmeyer, Christiane Kugler

**Affiliations:** 1 Institute of Nursing Science at the University of Freiburg, Freiburg, 79110 Germany; 2 Institute of Medical Biometry and Statistics, Faculty of Medicine and Medical Center – University of Freiburg, Freiburg, 79104 Germany; 3 Institute of Ethics, History and Theory of Medicine, Medical Faculty, Ludwig- Maximilians-Universität München, Munich, 80336 Germany

**Keywords:** Moral distress, Critical care nursing, Translation, Validation, German

## Abstract

**Background:**

Moral distress is an increasingly studied phenomenon in nursing science and ethics. It refers to the psychological discomfort experienced when an individual is prevented from acting on a course of action that they recognize as ethically correct. Nurses who work in intensive care are at high risk of experiencing moral distress.

Various instruments to assess moral distress, most recently Epstein et al.’s measure of moral distress for health care professionals (MMD-HP), have been developed. At present, there is no validated German language version of the instrument for assessing moral distress in Germany.

Therefore, this study aimed, at (1) translating and linguistically validating the instrument, as well as (2) psychometrically validating it among intensive care nurses in Germany.

**Methods:**

According to the ISPOR (International Society for Pharmacoeconomics and Outcomes Research) guidelines, the MMD‑HP was translated, linguistically validated, and psychometrically tested. The German version of the moral distress thermometer (MDT) and the German Nursing Workload Scale (NWS) were used to measure the construct validity of the translated instrument. Institutional ethics review boards of two universities approved the study protocols.

**Results:**

A total of 187 questionnaires of intensive care nurses remained for analysis after listwise deletion. The mean sum‑score was 106.9 (± 62) (minimum = 3, maximum = 325; range = 0–400). Criterion validity was supported by a high positive correlation between the German version of the MMD‑HP and the validated German-language moral distress thermometer (ρ = 0.598, p = 0.001, n = 168). Factor analyses revealed a four‑factor solution with correlating factors for the German version. The instrument showed high reliability (ωH = 0.91, 95 % CI = 0.88–0.93).

**Conclusions:**

Our findings demonstrate that the German adaptation of the MMD-HP shows potential for measuring moral distress among intensive care nurses in Germany. In addition, its psychometric properties preliminarily support its reliability and validity in this cultural context. Further independent validation studies are needed to provide support for the proposed four-factor model.

## Background

Over the last decade, the shortage of nurses has been one of the major challenges facing public healthcare services in Europe [1, 2]. Germany’s healthcare workforce comprises roughly 6 million professionals, including 1.25 million in hospital settings [3]. Within this group, intensive care units (ICU) employed approximately 28.000 specialized nurses as of 2020, according to the latest comprehensive dataset available [4]. No additional training is required to work in intensive care or intermediate care units (IMCU) in Germany.

Moral distress is playing an increasingly important role in nursing research and healthcare ethics [5–8], as it can relate to staff shortages, affect mental health, nurses well-being and the quality of care [7, 9–11]. When Jameton first introduced the term moral distress in 1984, he described it as a negative, distressing psychological state that arises when a person is aware of the ethically correct course of action in a specific situation, but is unable to implement it due to institutional or hierarchical constraints [12, 13]. Moral distress may create a psychological imbalance that can manifest itself in anger, frustration, guilt, worry, fear, helplessness, powerlessness, lack of self-esteem or depression [14]. Furthermore, it can lead to burnout, high staff turnover and exhaustion [5, 6, 15–17]. Moral distress remains a contested concept in the scholarly literature [18]. Two main definitions are commonly used. A broad formulation characterises moral distress as a general psychological response that can arise in any ethically challenging situation [19]. In contrast, the more precisely defined concept, originating with Jameton, describes moral distress as the specific psychological state that emerges when an individual recognises a moral imperative yet is constrained from acting on it [19, 20]. The present study adopts the latter definition, in line with the developer of the original MMD-HP instrument [21].

In the past, a variety of instruments to assess moral distress have been developed and validated for different target groups, as well as translated into multiple languages. These instruments include the moral distress thermometer [22], the moral distress scale (MDS) [23], the moral distress scale-revised (MDS-R) [24] and the measure of moral distress for healthcare professionals (MMD-HP) [25–29]. The German translation of the MDS by Kleinknecht-Dolf et al. [30] is an important contribution to the measurement of moral distress in the German-speaking context, yet it was concerned with Hamric & Blackhall’s version of Corley’s moral distress scale [31, 32]. Hamric & Blackhall adapted the MDS in order to be able to include nurses and physicians in their study, yet they did not provide information on the psychometric properties of their adapted scale [31]. We here provide a German translation of an updated instrument to measure moral distress, the MMD-HP [21]. Epstein et al. developed the MMD-HP based on the MDS-R [33] and recommended that the MMD-HP should replace the MDS-R. The items of the MMD-HP were carefully constructed to be usable by healthcare professionals in adult and pediatric critical, acute, or long-term acute care settings. The instrument was validated for two groups: nurses and physicians. The instrument reflects the common as well as most current causes of moral distress. The authors showed that the instrument has good psychometric properties and a clear factor structure (21). However, to date, there is no German-language version of the measure of moral distress for available for healthcare professionals. The aim of this study was therefore (1) translating and linguistically validating the instrument and (2) psychometrically validating a German version of the MMD-HP among intensive care nurses in Germany.

## Method

### Study design

The development of a German version of the MMD-HP was carried out in two phases: (1) translation and linguistic validation, and (2) piloting of the initially translated instrument, as well as psychometric validation using a cross-sectional study design [34, 35]. In this pilot study, intensive care nurses and nurses working in IMCU were included due to the fact that these are exposed to a high workload and responsibility and are faced with both emotionally and physically demanding tasks [11]. The determination of the sample size for this pilot study was based on the COSMIN checklist for patient-reported outcome (PRO) research [36].

The reporting of each phase was based on the Strengthening the Reporting of Observational Studies in Epidemiology (STROBE) Statement [37, 38]. The ethics committees of both participating sites (No. 21-16677), (23–0454 KB) approved the study protocol.

### Phase 1: translation and linguistic validation

**Fig. 1:**
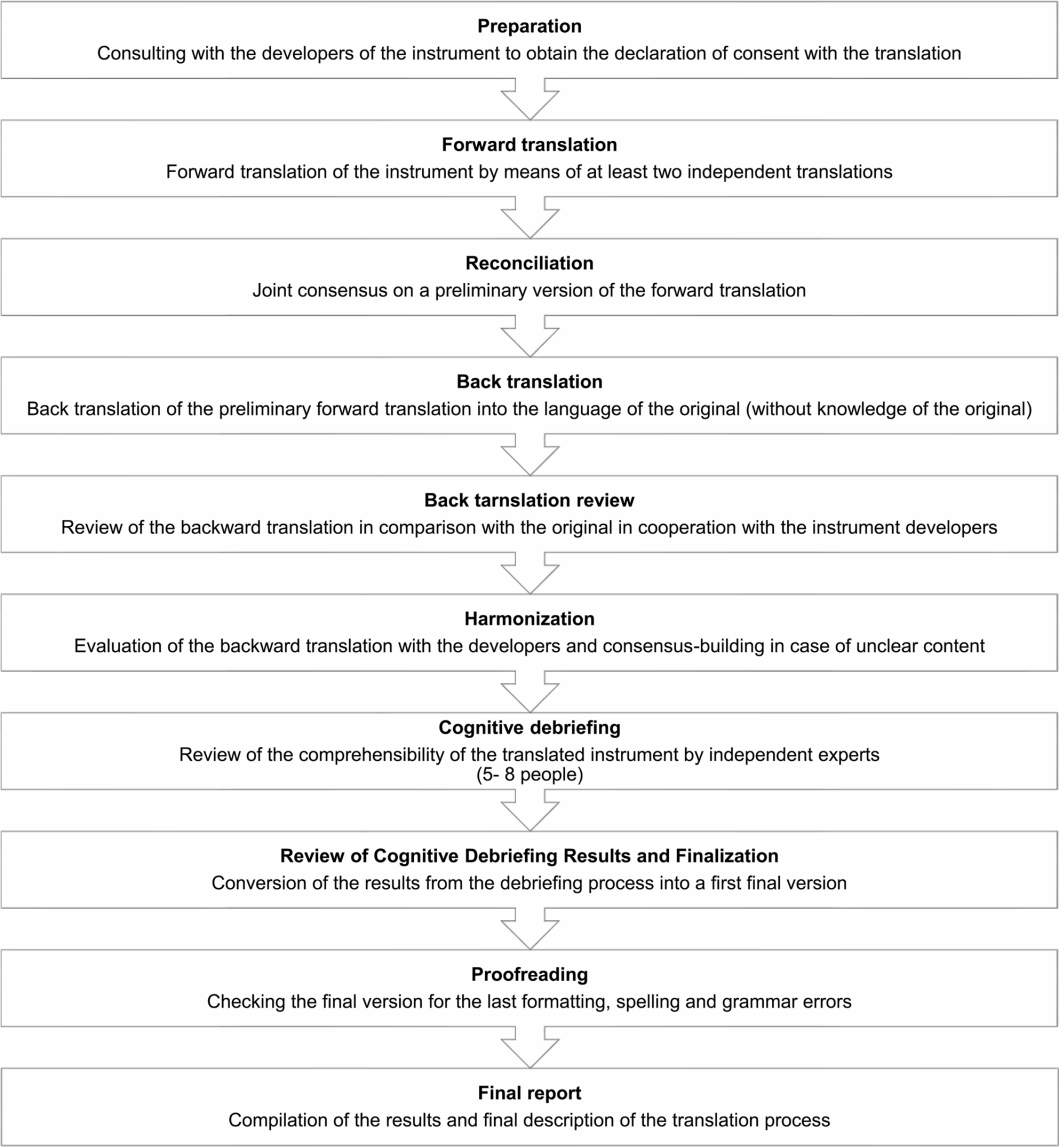
Self-created graphic of the linguistic validation using the ISPOR guideline [39]

The linguistic validation was conducted according to the International Society for Pharmacoeconomics and Outcomes Research (ISPOR) guidelines for translating and culturally adapting patient-reported outcome (PRO) instruments in clinical research [39]. The step-by-step process is depicted in Fig. 1. After obtaining written permission from the original English instrument developer Elizabeth G. Epstein, three independent forward translations were produced by native German speakers – including the first author and two colleagues. A committee consisting of three research associates, two of them with a background in nursing and nursing science and one with a background in sport science, who performed the forward translation synthesized these into a preliminary draft. During the backward translation, the preliminary German version of the instrument was translated back into English by two independent parties. Both parties were required to have no prior familiarity with the instrument [39]. Subsequently, together with the instrument’s developer, the two English versions were compared with each other within the framework of the harmonization process. To evaluate the instrument’s comprehensibility and relevance, a cognitive debriefing was performed in two successive testing cycles. For the calculation of the content‑validity index (I‑CVI), nursing experts employed in hospital settings, including intensive‑care units, were asked to rate each item’s linguistic clarity and relevance of each item to the measured construct using the following categories: *4 = highly relevant*,* 3 = quite relevant*,* 2 = somewhat relevant or 1 = not relevant at all*. Subsequently, responses were dichotomized: *Not relevant at all* and *somewhat relevant* were scored as 0 points [40, 41]. *Quite relevant* and *highly relevant* were scored as 1 point [40, 41]. The I-CVI for each item was then calculated by summing all points and dividing by the total number of expert ratings. An I-CVI ≥ 0.78 indicates “good” content validity for the item [40, 41]. Participant feedback from the debriefing was incorporated to refine wording, syntax, and terminology. A German linguistics specialist holding a master’s degree in German philology performed a final linguistic review, which included meticulous proofreading for grammatical and orthographic accuracy to guarantee precision.

### Phase 2: piloting and psychometric validation

#### Sample size and inclusion criteria

Intensive care nurses from two university hospitals in Germany were recruited between July 2023 and April 2024. We included both adult and pediatric ICUs (neonatology, paediatrics, surgical intensive care, and anaesthesiology intensive care) because both settings are known for being places where moral distress occurs rather frequently [6, 42]. The MMD-HP was developed for both care settings [21]. In site 1, all ICUs were invited to participate in the study, in site 2, we accessed healthcare staff on pediatric ICUs (including neonatal ICUs). Recruitment and data collection were carried out in both institutions by trained study personnel (LF, MS). Data entry and analysis took place at the leading site (SB). The following inclusion criteria were applied:

- Intensive care nurses (adult and paediatric units) working at one of the two university-based hospitals.
- At least three months working in an intensive or intermediate care unit.
- Sufficient German language skills to participate in this study.
- ≥ 18 years old.
- Written informed consent.

#### Instruments

##### MMD-HP

The MMD-HP is the result of extensive refinement and adaptation over time and was originally developed from the Moral Distress Scale (MDS) [21, 43]. The Moral Distress Scale (MDS) was developed in 2001 by Corley et al. to measure the frequency and degree of moral distress in intensive care nurses [43].

The MDS consists of 32 items and was designed using a 7-point Likert scale. The higher the value, the greater the burden of moral distress [43, 44]. The MDS was modified by Hamric et al. [9, 33, 44] in order to cover more situations that can cause moral distress for nurses as well as to include other healthcare professionals. The so-called Moral Distress Scale-Revised (MDS-R) was shortened to 21 items and information on frequency and degree of distress was assessed on a 5-point Likert scale. However, this instrument also had limitations, as it did not fully capture all causes contributing to the development of moral distress [21].

Therefore, Epstein et al. [21] reanalysed data from previous surveys using the MDS-R and identified additional factors contributing to the development of moral distress [21]. This overview led to the deletion of three items, the modification of three items, the consolidation of four items to two items and the addition of 11 items. The newly added items address causes of moral distress at the team and system levels, whereas the MDS-R only captured causes at the clinical level.

Epstein et al. developed an instrument that can be used by healthcare professionals in different settings. The instruments internal factor structure organizes its items into four domains of moral distress: (1) system‑level factors (2), patient‑level factors (3), team‑related factors concerning patients, and (4) team‑related factors concerning colleagues [21].

The MMD-HP captures the five main direct and indirect components of moral distress: complicity in misconduct, lack of voice, misconduct related to professional (not personal) values, repeated experiences, and three levels of causation (patient, unit, system). The instrument comprises 27 items and two open-ended questions that focus on leaving the profession and provides information on frequency and degree of distress using two 5-point Likert scales (higher numbers refer to more frequently occurring and a higher degree of moral distress). The frequency scale as well as the distress scale range from zero = never to four = very frequently. The frequency score (f) is multiplied by the degree of distress score (d) to obtain a composite score (fxd, range 0 to 16). The composite score therefore reflects the overall experience of moral distress, simultaneously representative of both frequency of event and the degree of distress. These scores are summated to obtain a MMD-HP sum score ranging from zero to 432, whereby the two open questions are not taken into account for the summation. Higher scores indicate higher levels of moral distress [21]. For the free text item “Are you currently thinking about leaving your job because of moral distress?“, the response options were expanded to include “I don’t know” alongside “Yes” and “No” to account for uncertainty in career decision-making.

##### Moral distress thermometer (MDT)

For the validation process of the MMD-HP, the German version of the MDT was used to measure construct validity of the translated instrument [22, 45]. The MDT was created by Wocial and Weaver [46] as a single-item tool designed to capture the intensity of moral distress as it occurs. Since its inception, the MDT has been employed worldwide to assess moral distress among health-care providers in a variety of settings, including intensive-care units [46].

##### Nursing workload Scale (NWS)

Besides the MDT, the NWS was used to measure the construct validity. The NWS measures the subjectively perceived workload of nurses based on motivation and stress theories. It includes 12 items across two dimensions: “coordination and information problems” and “psychophysical overload.” Responses are recorded on a five-level scale ranging from never to always [47]. Bartholomeyczik and associates defined any situation that blocks or impairs a motivational response as frustrating, producing a psychological tension called stress. Conceptually, potential stressors include work‑group disharmony, constrain, overload, or disturbed patient relationships. The model assumes that the cumulative effect of these stressors determines a person’s overall stress score, which in turn depends on the organization’s structure. Different reaction patterns are identified and linked to patient‑care behaviour. A lower organizational stress is associated with more positive outcomes for patients [47].

### Data collection

At the 1st site, recruitment and data collection was carried out by contacting all respective intensive care ward managers to present the topic in ward meetings. In addition, the link and QR code for the digital survey were passed on to potential participants. Following the respective ward meetings, the ward managers were sent an email with a brief presentation and information sheet. The digital survey at this site was administered through the REDCap™ survey system. Study staff at the 2nd site handled recruitment and conducted the survey either online or via pen and paper. The introduction to the survey provided participants with comprehensive information about the study, the inclusion criteria, and their rights under the EU General Data Protection Regulation (GDPR).

### Variables

For the psychometric validation the item and scale values of the described instruments as well as demographic data were analysed. The collected socio-demographic variables included, among others, the following variables: age (metric), gender (male/female/non-binary), highest professional qualification (registered nurse, registered nurse with additional training in anaesthesia and intensive care, registered nurse with a degree in nursing), type of hospital operator (state, church, and private) and satisfaction with the current professional situation (very dissatisfied to very satisfied).

### Statistical analyses

Confirmatory factor analysis (CFA), exploratory factor analysis (EFA) and internal consistency calculations were performed using the R statistical software (version 4.4.2, lavaan package). The remaining statistical analyses were conducted using IBM SPSS Statistics (version 29).

The construct validity of the MMD-HP GER was examined by testing the convergent and criterion-related validity. Convergent validity was based on hypothesis tests according to Abma et al. [48]. The responses to individual items were treated as interval-scale data for the analyses. The significance level was set at α = 0.05 (5.0%). The theoretical foundation for the validation was based on Classical Test Theory [49, 50]. A missing value analysis was performed for the MMD-HP items. Little’s test of missing completely at random (MCAR) [51] was performed to check whether the data were missing completely at random. The Kolmogorov-Smirnov test was used to test for normality. As this was a psychometric validation of a German version of an instrument that was defined a priori, a CFA was conducted to test the consistency of the data with the original model. Chi-square (χ2), chi-square/degrees of freedom (χ2/df), Comparative Fit Index (CFI), Tucker-Lewis Index (TLI), Root Mean Square Error of Approximation (RMSEA) and Standardized Root Mean Squared Residual (SRMR) were used to assess the goodness of fit. An EFA, using parallel analysis and oblique Promax rotation, was subsequently performed in order to reveal the underlying factor structure in the German sample in an exploratory manner. The reliability of the instrument was examined using McDonald’s Omega coefficients due to a violation of the essential Tau-equivalence for the calculation of Cronbach’s Alpha.

## Results

### Phase 1: linguistic validation

In the initial cognitive debriefing, twelve experts assessed the instrument, consisting of post‑graduate nursing students working in diverse clinical environments such as intensive care units. Five items fell below the acceptable threshold of 0.78 (scores: 0.50 and 0.75). Consequently, the translation committee revised these items before conducting a second debriefing. In the second cognitive debriefing, involving 17 post-graduate nursing students from the same cohort, all items met validation criteria, with I-CVI scores ranging from 0.88 to 1.0.

### Phase 2: psychometric validation

#### Characteristics of the study sample

The pilot study returned a total of 224 records. Of these, 37 were excluded on the basis of incompleteness (i.e. >30% of the data missing at case-level). The final dataset consisted of intensive care nurses from two German locations (location 1: *n* = 100; location 2: *n* = 87). Table 1 depicts the proportion cases with missing data on instrument level.

A missing value analysis was performed for the MMD-HP items. Missing value analysis, using Little’s MCAR test, confirmed data were missing completely at random on item-level. Furthermore, an in-depth analysis of MMD-HP items with more than 5% missing values (items 13–27) revealed no relevant associations in the missing data indications in relation to the socio-demographic characteristics of the sample, justifying listwise deletion for further analysis using the MMD-HP.

**Table 1:** Missing data on instrument-level (*n* = 187)

Instrument	*N* complete	Missing (%)
Survey Total (all items)	115	38.5
Socio-demographic and occupational items	153	18.2
MMD-HP GER (based on calculated composite item scores)	147	21.4
Moral Distress Thermometer	168	10.2
Nursing Workload Scale	165	11.8

The sample characteristics of participants can be found in Table 2.

**Table 2:** Socio-demographic data of the sample of the psychometric validation (*n* = 187)

Variable	Frequency (%) or mean (SD)
*Socio-demographic data*	
Gender	
Female	152 (81.3%)
Male	34 (18.2%)
Non-binary	1 (0.5%)
Missing	0 (0%)
Age	38.63 (±10.7)
Missing	8 (4.3%)
Professional qualification	
Registered Nurse (RN)	82 (43.9%)
RN with additional training in anesthesia and intensive care	88 (47.1%)
Completed degree in nursing science	15 (8.0%)
Missing	2 (1.1%)
Number of years in the profession	15.71 (±10.48)
Missing	9 (4.8%)
Number of years in intensive care	13.59 (±10.08)
Missing	6 (3.2%)
*Professional situation*	
Type of hospital operator	
State	182 (97.3%)
Church	0 (0.0%)
Private	4 (2.1%)
Missing	1 (0.5%)
Current focus of work	
Direct bed-side patient care	167 (89.3%)
Management	15 (8.0%)
Other	4 (2.1%)
Missing	1 (0.5%)
Satisfaction with the current professional situation	
Very dissatisfied	2 (1.1%)
Dissatisfied	23 (12.3%)
Not very dissatisfied	14 (7.5%)
Neither satisfied nor dissatisfied	31 (16.6%)
Not very satisfied	23 (12.3%)
Satisfied	85 (45.5%)
Very satisfied	8 (4.3%)
Missing	1 (0.5%)

### Psychometric properties

#### Item analysis

**Fig. 2:**
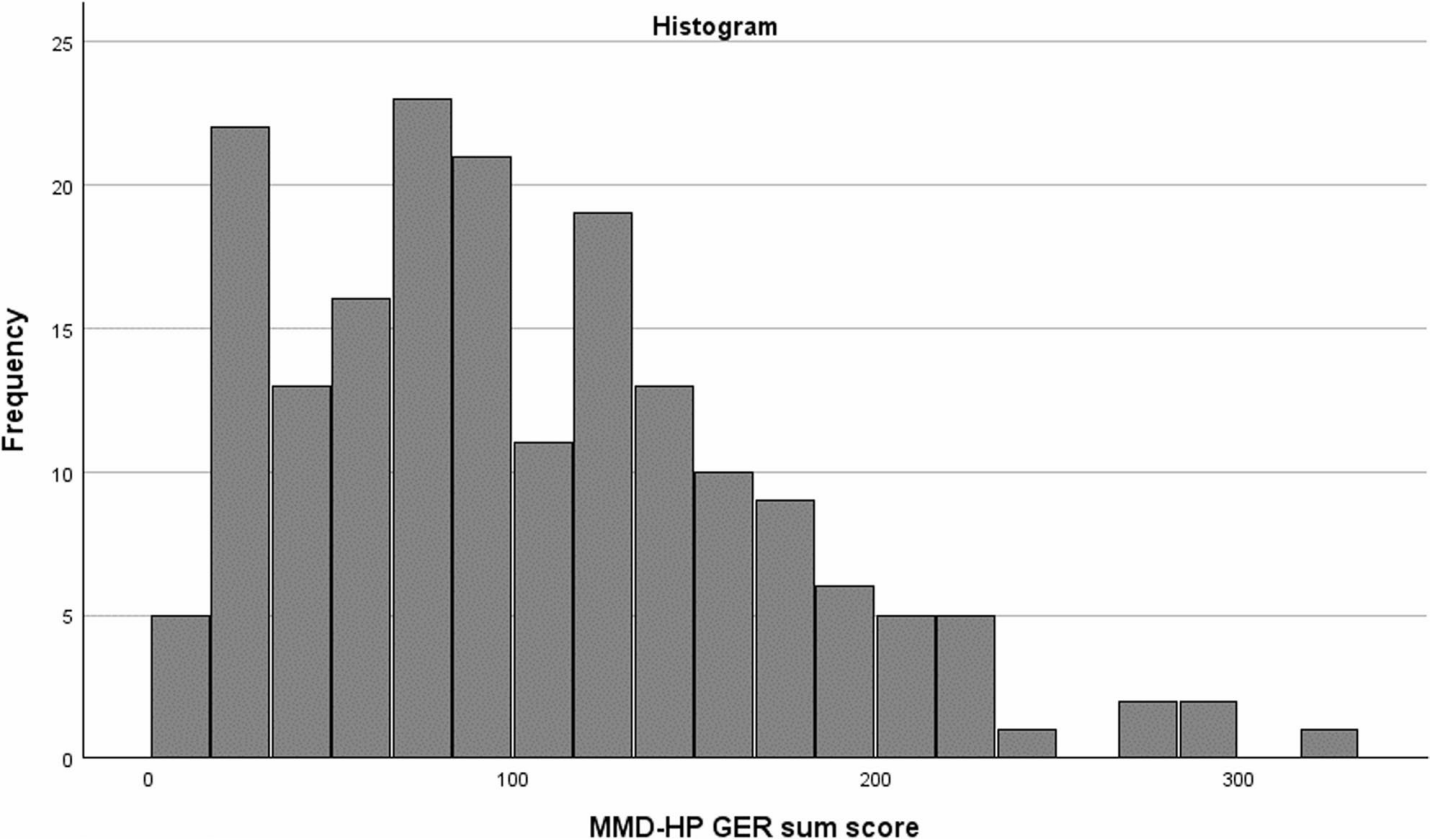
Distribution of MMD-HP GER sum score with 27 items (total possible range 0-432; higher scores indicate higher moral distress, *n* = 147)

The average MMD-HP sum score of the 147 valid surveys was 106.9 (± 62.5), with a minimum score of 3 and a maximum score of 325. The median score was 93. The test score distribution is right-skewed (skewness = 0.8). Figure 2 shows the distribution of the MMD-HP GER sum score. The Kolmogorov-Smirnov Test on normal distribution confirmed the non-normally distributed sum score values.

The highest numbers of valid values were *n* = 187 for items 1, 2, and 3. From item 13 to 27, at least 7.5% of the values were missing. The full range (0–16) of response options was used for all items, except for item 27 (0–12). Floor and ceiling effects were identified if more than 50% of the frequency of responses were 0 or 16. There was a floor effect for a total of six items. The frequency of the minimum value (0) was at or above 50% for the items 6, 11, 12, 20, 21 and 27. All items had a high difficulty, with a range of 5.4% to 49.3%. The average item difficulty for the entire instrument was P_total_= 24.7%. A total of eleven items with low difficulty indices below the index range of 20% were identified. All other item difficulty indices were in the middle index range. Item 17 was the item with the lowest difficulty, P_fxd__17 = 49.3%. The discriminatory potential of the items was positive between 0.3 and 0.6. The individual items therefore make a moderate contribution to the measurement accuracy and validity of the instrument. Table 3 shows the psychometric properties of the MMD-HP GER.

**Table 3:** Psychometric properties of the MMD-HP GER

Item	Median	Mean value (± SD)	Variance	Min. - Max. (span)	IQ range	Skew (SE)	Excess (SE)	*P*_i_ (%)	*r*_i(x−i)_
*N* valid = 147									
1	6.00	5.49 (± 4.288)	18.39	0–16 (16)	7	0.667 (0.2)	-0.233 (0.397)	34.31	0.464
2	3.00	4.43 (± 4.620)	21.34	0–16 (16)	8	0.876 (0.2)	-0.148 (0.397)	27.68	0.449
3	4.00	4.50 (± 4.225)	17.85	0–16 (16)	5	1.145 (0.2)	0.731 (0.397)	28.15	0.583
4	1.00	3.56 (± 4.941)	24.41	0–16 (16)	6	1.414 (0.2)	0.801 (0.397)	22.28	0.463
5	1.00	3.47 (± 4.533)	20.55	0–16 (16)	6	1.333 (0.2)	0.753 (0.397)	21.68	0.466
6	0.00	1.61 (± 3.411)	11.64	0–16 (16)	1	2.677 (0.2)	6.956 (0.397)	10.08	0.396
7	1.00	2.45 (± 3.556)	12.65	0–16 (16)	3	1.764 (0.2)	2.433 (0.397)	15.31	0.289
8	6.00	6.24 (± 4.888)	23.89	0–16 (16)	7	0.521 (0.2)	-0.781 (0.397)	38.99	0.577
9	6.00	6.86 (± 4.720)	22.27	0–16 (16)	5	0.409 (0.2)	-0.774 (0.397)	42.86	0.617
10	1.00	2.78 (± 3.575)	12.78	0–16 (16)	4	1.370 (0.2)	1.300 (0.397)	17.35	0.373
11	0.00	2.59 (± 4.128)	17.04	0–16 (16)	4	1.767 (0.2)	2.231 (0.397)	16.20	0.564
12	0.00	0.86 (± 2.714)	7.36	0–16 (16)	0	3.975 (0.2)	16.524 (0.397)	5.40	0.396
13	6.00	6.65 (± 5.086)	25.87	0–16 (16)	10	0.431 (0.2)	-0.917 (0.397)	41.58	0.573
14	6.00	7.09 (± 4.719)	22.27	0–16 (16)	5	0.443 (0.2)	-0.721 (0.397)	44.30	0.599
15	1.00	3.08 (± 4.394)	19.31	0–16 (16)	4	1.732 (0.2)	2.194 (0.397)	19.26	0.537
16	6.00	6.29 (± 5.025)	25.25	0–16 (16)	7	0.636 (0.2)	-0.705 (0.397)	39.29	0.557
17	6.00	7.89 (± 5.685)	32.32	0–16 (16)	10	0,190 (0.2)	-1,398 (0.397)	49.32	0.450
18	3.00	4.73 (± 5.127)	26.28	0–16 (16)	8	1.006 (0.2)	-0.150 (0.397)	29.55	0.582
19	2.00	4.03 (± 4.840)	23.42	0–16 (16)	6	1.272 (0.2)	0.571 (0.397)	25.17	0.501
20	0.00	1.71 (± 3.296)	10.87	0–16 (16)	2	2.912 (0.2)	9.026 (0.397)	10.67	0.333
21	0.00	1.08 (± 2.663)	7.09	0–16 (16)	1	3.343 (0.2)	11.985 (0.397)	6.76	0.349
22	2.00	3.80 (± 4.546)	20.67	0–16 (16)	6	1.399 (0.2)	0.989 (0.397)	23.72	0.510
23	1.00	2.20 (± 3.474)	12.07	0–16 (16)	4	2.106 (0.2)	4.221 (0.397)	13.78	0.492
24	3.00	4.60 (± 4.804)	23.08	0–16 (16)	5	1.042 (0.2)	0.035 (0.397)	28.74	0.622
25	4.00	4.46 (± 4.524)	20.47	0–16 (16)	5	1.269 (0.2)	0.792 (0.397)	27.89	0.555
26	2.00	3.07 (± 3.823)	14.61	0–16 (16)	4	1.834 (0.2)	3.365 (0.397)	19.18	0.498
27	0.00	1.37 (± 2.333)	5.44	0–12 (12)	2	2.407 (0.2)	6.412 (0.397)	8.59	0.271
MMD-HP Score	95	106.89 (± 62.492)	3.905.262	3–325 (322)	82	0.805 (0.200)	0.712 (0.397)	24.74	

*SD*  Standard deviation, *IQ range*  interquartile range, *SE*  standard error, *P*_*i*_ item difficulty, *r*_*i(x−i)*_ item discrimination

#### Validity

Convergent validity showed a high positive correlation between the total score of the MMD-HP GER and the nursing workload scale (ρ = 0.701, *p* < 0.001, *n* = 168). Similarly, a high positive correlation was identified for criterion validity between the MMD-HP GER and the validated German-language moral distress thermometer (ρ = 0.598, *p* = 0.001, *n* = 168).

#### Confirmatory factor analysis with the original instrument model

The CFA tested the pilot data on a higher-order model with four non-correlated factors, as proposed by Epstein et al. [21]. The chi-square statistic (χ2 = 708.642, *p* < 0.001) indicated significant deviation from the proposed model. The χ2/*df* value of 2.221 is slightly above the commonly suggested ‘good’ cut-off value of 2 for good fit [35]. In the original model [21], item 22 exhibited cross-loadings on two factors: “system root-causes” (0.493) and “clinical root-causes” (0.422). In contrast, in our study the item demonstrated a moderate factor loading on “system root-causes” (0.505) and minimal loading on “clinical root-causes” (0.003). Multicollinearity was found to be present. As a result, an EFA was subsequently conducted to determine the translated instrument’s internal structure in an exploratory manner. The current model’s fit statistics fall short of the recommended cut-offs (Comparative Fit Index (CFI) = 0.751; Tucker-Lewis Index (TLI) = 0.725), indicating that the model requires further refinement (Table 4).

**Table 4:** Quality criteria of the confirmatory factor analysis with the original instrument model

Quality criterion	Results (robust)	Good model fit	Acceptable model fit
*χ2-value* (p-value)	592.803 (*p* < 0.001) [Yuan-Bentler correction] Model does not fit the model-implied covariance matrix		
*χ2-value* (χ2/df)	2.221	*χ2/df* ≤ 2	χ2/df ≤ 3
RMSEA	0.084	RMSEA ≤ 0.05	RMSEA ≤ 0.08
CFI	0.751	CFI ≤ 0.97 (homogeneous items) CFI ≤ 0.95 (heterogeneous items)	CFI ≤ 0.95 CFI ≤ 0.90
TLI	0.725	TLI ≤ 0.97 (homogeneous items) TLI ≤ 0.95 (heterogeneous items)	TLI ≤ 0.95 TLI ≤ 0.90
SRMR	0.091	SRMR ≤ 0.05	SRMR ≤ 0.10

*χ2-value*  Chi-square value, *df*  degree of freedom, *RMSEA*  root mean square error of approximation, *CFI*  comparative fit index, *TLI*  Tucker-Lewis Index, *SRMR*  standardized root mean square residual

#### Exploratory factor analysis

**Fig. 3:**
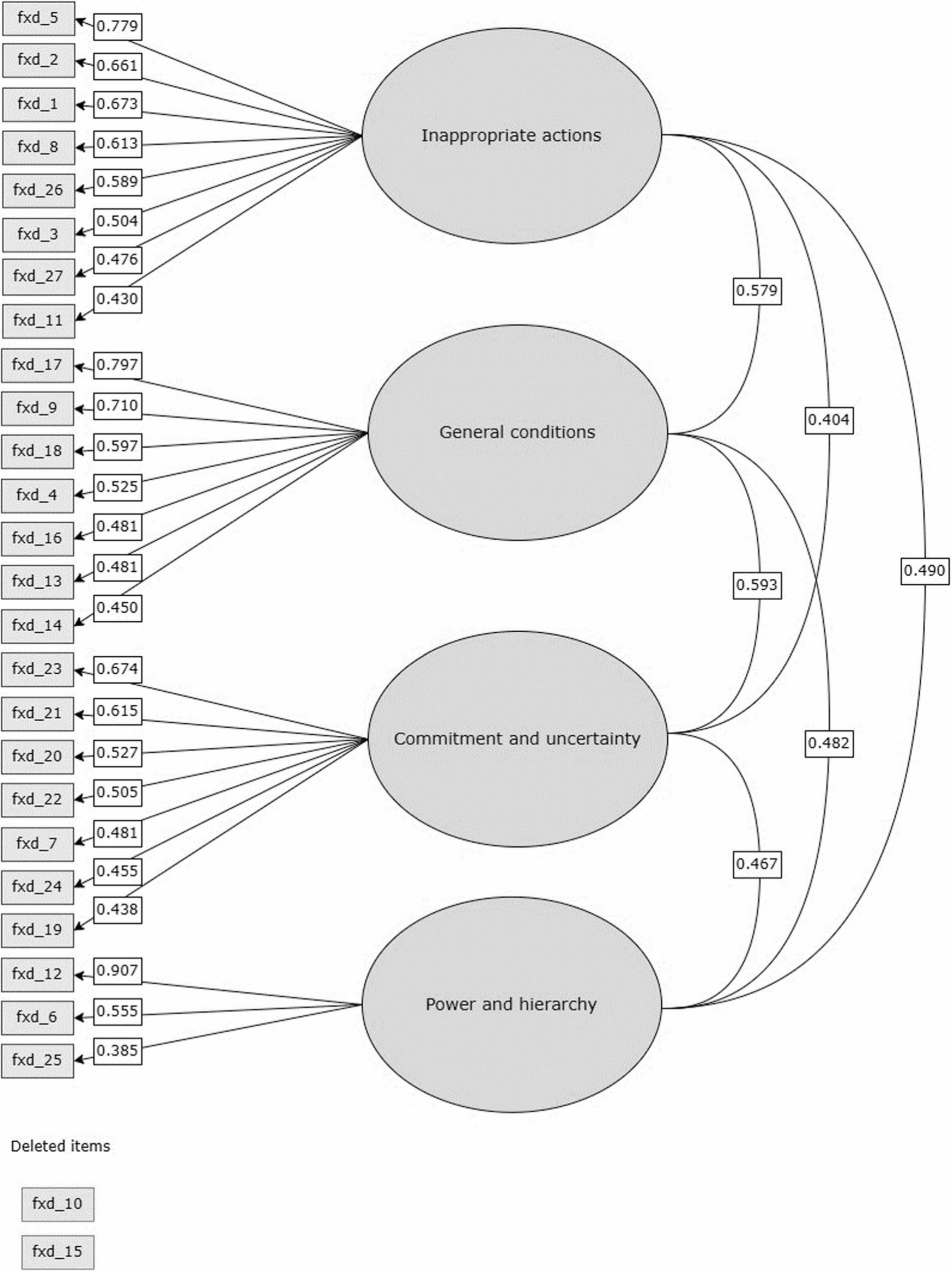
The four factors of the MMD-HP GER

An EFA was conducted to determine the instrument’s factor structure within the German setting. The parallel analysis identified a four-factor structure with correlating factors for the German version of the MMD-HP, explaining 41.6% of the variance. Model fit indices were RMSEA = 0.068, TLI = 0.995 and SRMR = 0.091. The factor loadings ranged from 0.385 to 0.907, with some double loadings on factors. Through a qualitative post-hoc interpretation with five experts, the four factors were labelled and agreed upon through content-analysis method (Fig. 3). The items loaded onto four factors in different item constellations to the original model. Items with double factor loadings were taken into account but removed after reconciliation. Two items were excluded which was in accordance with the cut-off value of > 0.3.

#### Confirmatory factor analysis with the observed pilot data

A confirmatory factor analysis was carried out to preliminarily test the fit of the pilot data to the model determined by the exploratory factor analysis, namely with 25 items and a 4-factor structure with correlating factors.

**Table 5:** Quality criteria of the confirmatory factor analysis of a 25-item 4-factor model with correlating factors

Quality criterion	Results (robust)	Good model fit	Acceptable model fit
*χ2-value* (p-value)	453.695 (*p* < 0.001) [Yuan-Bentler correction] Model does not fit the model-implied covariance matrix		
*df*	269		
*χ2-value* (χ2/df)	1.697	*χ2/df* ≤ 2	χ2/df ≤ 3
RMSEA	0.075	RMSEA ≤ 0.05	RMSEA ≤ 0.08
CFI	0.814	CFI ≤ 0.97 (homogeneous items) CFI ≤ 0.95 (heterogeneous items)	CFI ≤ 0.95 CFI ≤ 0.90
TLI	0.793	TLI ≤ 0.97 (homogeneous items) TLI ≤ 0.95 (heterogeneous items)	TLI ≤ 0.95 TLI ≤ 0.90
SRMR	0.082	SRMR ≤ 0.05	SRMR ≤ 0.10

*χ2-value*  Chi-square value, *df*  degree of freedom, *RMSEA*  root mean square error of approximation, *CFI*  comparative fit index, *TLI*  Tucker-Lewis Index, *SRMR*  standardized root mean square residual

The CFA for a model with correlated factors exhibited an overall improved model fit (Table 5) in comparison to the original higher-order model proposed by Epstein [21] with a range of factor loadings from 0.363 to 0.738.

### Reliability

McDonald’s Omega for subscale totals was calculated for the new factors (inappropriate actions = 0.833, general conditions = 0.821, commitment and uncertainty = 0.786, power and hierarchy = 0.641). The MMD-HP GER showed high reliability (ωH = 0.91, 95% CI = 0.88–0.93).

## Discussion

To the best of our knowledge, this is the first pilot study, which translated and attempted to validate the MMD-HP into German. In light of increasing reports of moral distress in the German healthcare system [11] data collected based on this instrument may inform and guide design interventions to reduce moral distress in ICU nurses. The results of this study demonstrate that the German adaptation of the MMD-HP offers promising indications for successful future validation studies with the proposed model for assessing moral distress in Germany. Psychometric evaluation indicated good measurement properties, supporting its use in hospital settings. During the linguistic validation process, items were culturally adapted to reflect the specific activities and contextual realities of German intensive care practice. Subsequent factor analysis revealed a refined four-factor structure, with intercorrelated dimensions that enhanced the instrument’s applicability to the German healthcare context. Thus, our findings serve as a constructive baseline for improving the model’s alignment with empirical data, guiding future adjustments to better capture the underlying factor structure. Our findings should be interpreted as providing preliminary evidence for reliability and validity, nevertheless requiring further independent validation in a new sample in future studies.

Our findings indicate that German participants reported higher levels of moral distress compared to the Swedish or Japanese validation studies [25, 26]. The sum score of the Swedish study ranged from 6 to 193, with a sum score of 67.81 (± 42.22). The Japanese validation study yielded a sum score of 98.2 (± 59.9) with a range from 0 to 432. Both studies included nurses, physicians as well as physio- or occupational therapists [25, 26]. The Spanish version showed a sum score of 128.5 (± 70.9) with a range of 0–424; including nurses and physicians [28]. Among the German participants, the total sum score was 106.9 (range = 3–325; median = 93), indicating higher distress levels among participants. Opportunities for a direct comparison are limited, since the other studies involved physicians as well as further settings. Petersen and Melzer surveyed German home‑care nurses (*n* = 976) to identify work‑related predictors and outcomes of moral distress. Using the MDS and Copenhagen Psychosocial Questionnaire (COPSOQ III), they found that high emotional demands, frequent work‑life conflicts, low autonomy and weak social support increased distress, and that limited time for patient care further amplified it. Elevated distress was associated with higher burnout, poorer self‑rated health, and a greater intention to quit, but not with increased sickness absence [52]. Mehlis et al. examined the frequency and intensity of moral distress among oncology physicians and nurses facing decisions to limit life‑prolonging treatment. In a prospective study at a German university hospital, 100 advanced‑cancer inpatients undergoing such limitations were enrolled, and their treating physicians and nurses completed the MDT plus an open‑ended question on distress sources. Moral distress was reported by 74% of nurses (*n* = 67) with a mean distress score of 2.3 (± 2.3) [22]. The determination of the sample size for our pilot study was based on the COSMIN checklist for patient-reported outcome (PRO) research [36].The COSMIN checklist includes ten domains, each scored on a 4-point scale derived from its risk-of-bias tool. The scale serves as a guide for interpreting the methodological impact of study design choices. For content-validity testing of patient-reported outcome measures (PROMs), the checklist advises that each item be assessed by a sufficient number of participants [36]. In agreement with the COSMIN guideline the sample size was set to more than 50 participants in the pilot phase. During the psychometric validation of the MMD-HP GER, an EFA was conducted following the CFA due to the suboptimal fit between the original model [21] and our empirical data. The original factor structure (21) was not empirically confirmed through CFA in prior studies [28], which further justified our decision totake an exploratory approach with the pilot data to assess its psychometric properties in the German sample. As part of the translation and linguistic validation following the ISPOR guidelines, a cultural adaptation was performed. Cultural adaptation already starts during the translation steps, but it is carried out formally and systematically during harmonization and cognitive debriefing [39]. Both steps ensure that the instrument is not only linguistically accurate but also conceptually and experientially appropriate for the target culture [39]. Items 10 and 15 were removed due to not meeting the cut-off value of > 0.3 [35, 53] and due to their lack of relevance for the German context [54]. Item 10 (“follow a physician’s or family member’s request not to discuss the patient’s prognosis with the patient/family”) assumes that intensive care nurses are able to withhold important information regarding the patient’s health. By law, healthcare professionals, especially physicians, are required to inform patients about their prognosis [55, 56]. Second, in Germany, information about health conditions and prognoses is communicated only by physicians to patients and their relatives [55, 56]. Item 15 (“feel pressured to ignore situations in which patients have not been given adequate information to ensure informed consent”) was omitted due to the fact that the patient rights act requires that the treating physician clearly explains the diagnosis, findings, therapeutic options, and prognosis to the patient. Normally nurses are not involved and therefore unable to assess whether the information was complete. The existing 25 items were assigned to a total of four new main categories via the exploratory factor analysis, which could be interpreted as the main triggers for moral distress in the German context: inappropriate actions, general conditions, obligation and uncertainty and, finally, power and hierarchy. The lower Omega-value for power and hierarchy could be attributed to the lower number of items in this factor. For the purposes of providing preliminary results on validity and reliability for the proposed German model, a CFA was conducted using the pilot data. Performing a CFA on the same sample increases the risk of overfitting and can lead to inflated reliability estimates. For this reason, the reliability estimates provided should be interpreted with caution [34].

The revised factor structure identified in our study carries significant implications for future use of the instrument. First, the divergence from the original model [21]; the emergence of four distinct factors in the German context suggests that subscale-level analyses may be more appropriate than an overall sum score calculation. This is especially relevant given the cultural and systemic differences between healthcare settings in Germany and those in the original validation sample. In the German healthcare context, the scope of practice for nursing professionals differs significantly from that in the USA, particularly regarding autonomous decision-making in clinically complex or ethically sensitive situations [57]. In Germany, such decisions fall exclusively under physician-led authority [58] with nurses limited to advocacy, documentation, or escalation to medical staff rather than independent action. Furthermore, our findings underscore the necessity of cross-cultural validation before applying an instrument in new contexts. Future studies should prioritize:

- evaluating the factor structure in larger, representative German samples to confirm the stability of the four-factor structure.
- developing subscale evaluation tailored to the German context, which could enhance the instrument’s diagnostic utility for targeted interventions (e.g., addressing systemic vs. clinical root causes).

This pilot adaptation not only aligns the instrument with the realities of German healthcare practice but also opens avenues for comparative research on how structural and clinical factors differentially impact outcomes across international settings. The use of the MMD-HP GER can offer the opportunity to develop tailored interventions by recording moral distress in the setting of intensive and intermediate care nurses on paediatric and adult wards. With the help of interventions, the occurrence of moral distress and turnover in the profession holds potential to be counteracted.

### Limitations

This study has limitations. One relevant aspect is that the original instrument by Epstein et al. was validated for healthcare professionals such as nurses, physicians, and other health care clinicians working in acute care, intensive care, the operating room, emergency department, long-term acute care hospital, or outpatient clinic settings [21]. In contrast, the German version of the MMD‑HP (MMD‑HP GER) was validated solely with intensive care nurses. A second relevant aspect is the possible response fatigue of the participants towards the end of the survey. From items 13 to 27, at least 7.5% of the values were missing. This could explain the occurrence of missing values, as the motivation to fully answer the items could decrease over the course of the survey. Studies show that cognitive fatigue and decreasing engagement over the duration of a questionnaire can lead to incomplete or inconsistent responses [59]. Third, another potential influencing factor is recall bias. As participants were asked to refer to events from the last three months, there is a possibility that the memory of certain events is distorted or incompletely recalled. People tend to perceive and remember past experiences selectively, which can lead to an over- or underestimation of certain aspects of workload. This should be taken into account when interpreting the results [60]. A fourth important aspect concerns the timing and context of data collection. The evaluation of the participation times at the 1st site indicates that the questionnaires were completed during working hours. This could have both positive and negative effects on data quality. On one hand, participation during working hours could help to ensure that the answers better reflect the current work situation and attitudes towards it. On the other hand, there is a risk that external factors such as time pressure or work interruptions may affect participants’ concentration and diligence [61]. Despite their importance, surveys in the healthcare sector appear to be characterized by a declining number of participants. The reported response rates for surveys among nursing staff are now generally below 60% [61]. Fifth, as part of the translation and linguistic validation process, students participated in the cognitive debriefing. Although they had clinical nursing expertise and some ICU exposure, they were not exclusively working in intensive care, which may have limited the transferability of the results to this target group. In addition, there was a power relationship between the researcher and the students, which could potentially have influenced the openness and honesty of the feedback [62]. This limitation should be taken into account when interpreting the results. Sixth, the data collection at the 1st site was administered through the REDCap™ survey system, whereas at the 2nd site, study staff administered the questionnaire in pen‑and‑paper format to the paediatric intensive care nurses. Although web‑based and pen‑and‑paper surveys each have clear benefits, the question of whether they yield comparable results remains unresolved [63]. Several studies have found no substantive differences between the two formats [64, 65], whereas other research reports mode‑specific discrepancies [66, 67]. These findings are largely attributed to differences in administration context: pen‑and‑paper questionnaires are usually completed together in a controlled environment, while web‑based questionnaires are answered privately, at different times, and on a variety of devices. Both modes demonstrate acceptable test‑retest reliability yet systematic assessments of measurement invariance across administration contexts have not been conducted [68]. This limitation must be considered when interpreting the findings. Our study also carries some strengths. First, the translation and linguistic adaptation process very rigorously followed the guideline steps recommended by the ISPOR group [39]. In both cognitive debriefings, postgraduate nursing science students from the same cohort were involved. Beaton et al. emphasize that the same participants are preferred for pre‑testing to allow direct comparison of item performance [69].

Second, data was collected at two different sites by trained data collection staff. Third, in order to capture moral distress in all relevant nurse sub-groups, all nurses working at intensive or intermediate care wards and those caring for paediatric or adult patients were invited to participate and to present the entire diversity of this nurse specialty.

## Conclusion

Our results demonstrate that the proposed model for the MMD‑HP GER can reliably assess moral distress in German ICU nurses, and retains the psychometric robustness of the original English instrument [21]. Further independent validation studies are needed to provide additional evidence for the proposed model. By following ISPOR guidelines, the instrument captures German‑specific nuances while remaining directly comparable to international studies, facilitating cross‑cultural benchmarking and multinational research [25–29, 39].

## Data Availability

The datasets used and analysed during the current study are available from the corresponding author on reasonable request.

## Abbreviations

MMD-HP: Measure of moral distress for healthcare professionals
ISPOR: The Professional Society for Health Economics and Outcomes Research
ICU: Intensive care unit
IMC(U): Intermediate Care (Unit)
STROBE: Strengthening the Reporting of Observational Studies in Epidemiology
PRO: Patient-reported outcome
I-CVI: Content validity index
MDS: Moral distress scale
MDS-R: Moral distress scale-revised
MDT: Moral distress thermometer
NWS: Nursing workload scale
GDPR: General Data Protection Regulation
CFA: Confirmatory factor analysis
MCAR: Missing completely at random
CFI: Comparative fit index
TLI: Tucker-Lewis index
RMSEA: Root mean square error of approximation
SRMR: Standardized root mean squared residual
EFA: Exploratory factor analysis
USA: United States of America
COPSOQ III: Copenhagen Psychosocial Questionnaire
COSMIN: Consensus-based Standards for the selection of health status Measurement Instruments
